# Commentary: The impact of 24-h shift on cardiac anesthesia fellows

**DOI:** 10.3389/fpubh.2026.1942503

**Published:** 2026-10-12

**Authors:** Thibault Vieille, Gaël Piton

**Affiliations:** 1 Medical Intensive Care Unit, Besançon University Hospital, Besançon, France; 2 Universite Marie et Louis Pasteur, Besançon, France

**Keywords:** physician, shift, sleep disorder, work organization, work-related stress

We thank Liu et al. (1) for their interest in sleep disorders among physicians exposed to shift work. Over the last years, increasing attention has focused on work-related psychosocial factors.

Liu et al. showed that extended 24-h shifts among anesthesiologists resulted in significant sleep deprivation with an average self-reported sleep duration of 6.25 h and an average wristband-measured total sleep duration of 286.9 min (4.8 h). Extended 24-h shifts were associated with higher post shift fatigue scores (Profile of Mood States) and increased post-shift sleepiness levels (Karolinska Sleepiness Scale).

However, the effects of extended 24-h shifts may extend beyond the immediate post-shift period and lead to chronic sleep impairment. To explain this, several authors have suggested that night shift induced circadian misalignment and impaired melatonin secretion (2).

We hypothesize that work-related stress may act as a key mediator linking extended 24-h shifts to impaired off-shift sleep quality among physicians.

Indeed, in a cross-sectional survey of 218 French intensivists from 38 ICUs, the prevalence of poor sleep quality (Pittsburgh Sleep Quality Index > 5) was 58%. In multivariate analysis, poor sleep quality was associated with higher perceived stress intensity at work, as measured by the recently developed PS-ICU scale, which was specifically designed for ICU professionals (3).

Shift work leads to the depletion of physiological reserves: unhealthy diet (rushed eating, inadequate water intake, and excessive sugar intake), reduced sleep duration, and extensive walking. The effects of shift work on the autonomic nervous system (increased stress hormone levels, sympathetic activation), as reported by Rauchenzauner et al. (4) constitute an integrated marker of this physiological strain.

Shift work also contributes to the depletion of mental resources. Physicians must cope with high emotional and cognitive demands. According to Karasek's job strain model, psychological strain experienced during a shift results from the interaction between high job demands and low job control (5):

1. high job demands: uncertainty, task organization and prioritization, increased vigilance, high workload, challenging interactions with patients and their families, fear of clinical errors, ethically challenging situations.
2. low job control: limited control over workload, unit organization, difficulties in managing break times, staff shortages, frequent task interruptions.

Work-related stress is associated with impaired sleep as it induces increased physiological and psychological activation incompatible with sleep initiation and normal sleep patterns (6). We emphasize that a vicious cycle may develop, whereby stress impairs sleep, while fatigue further increases stress reactivity and individual susceptibility to stress.

We believe that it is urgent to move away from extended 24-h shifts for physicians working on-call shifts. We owe it to physicians, for their mental and physical health. A review by Hulsegge showed that limiting shifts to 16 h increased off-shift sleep duration and reduced sleepiness during shifts (Karolinska Sleepiness Scale) (7). The extended duration of medical shifts often raises concerns among the public. Other high-risk professions such as airline pilots for example are subject to stringent working-time regulations to ensure passenger safety. Recent guidelines from the French Society of Intensive Care (8) recommend a maximum of 16 h of consecutive work but they encounter resistance, especially because of concerns about disrupting continuity of care, high frequency of shifts, and changing established practices.

However, in order to ensure safety and quality of care, a reduction in shift duration should not be associated with an increased workload or a decline in the quality of handoffs as these could otherwise offset the potential benefits (9).

It may be appropriate to reassess the work organization from an ergonomic perspective, as suggested by Costa et al. (10): protected rest periods during shifts, the use of naps, development of personal coping strategies, a transition to day shifts after reaching a certain age. Compensation must not be purely monetary: it should also include additional compensatory rest days between two shifts, and improvements to the working environment. The health of physicians working on-call shifts should be regularly monitored by occupational health professionals. We support the implementation of 12-h shifts and the expansion of the medical workforce. In parallel, strategies aimed at reducing stress among physicians should be implemented including relaxation techniques, physical exercise, and limiting the consumption of stimulants.

We must implement a system that ensures continuous medical presence for patients while sustaining the medical workforce in the short, medium and long term.
